# Fracture Resistance of Root Canals Obturated with Gutta-Percha versus Resilon with Two Different Techniques

**Published:** 2013-08-01

**Authors:** Hengameh Ashraf, Golnaz Momeni, Nima Moradi Majd, Hamed Homayouni

**Affiliations:** a Department of Endodontics, Shahid Beheshti University of Medical Sciences, Tehran, Iran; b Dental Research Center, Research Institute of Dental Sciences, Shahid Beheshti University of Medical Sciences, Tehran, Iran; c Department of Endodontics, Dental Carries Research Center, Dental School, Qazvin University of Medical; d Iranian Center for Endodontic Research, Research Institute of Dental Sciences, Shahid Beheshti University of Medical Sciences, Tehran, Iran

**Keywords:** Endodontics, Resilon Sealer, Root Canal Therapy, Tooth Fractures

## Abstract

**Introduction:**

Dentin removal during root canal instrumentation creates a weaker root structure and increases its potential to fracture. The aim of this in vitro experimental study was to compare fracture resistance of teeth filled with gutta-percha, and Resilon using two different techniques.

**Materials and Methods:**

This study was performed on 105 single-canal extracted maxillary incisors. Samples were divided into seven groups of 15 each. Three groups were prepared with K-files; three groups with Race rotary files and in one group no preparation was carried out. Of all samples prepared either manually or with rotary instruments, 15 teeth were obturated using gutta-percha and AH26 sealer, 15 teeth were filled with Resilon and 15 teeth remained unfilled. Loading force to fracture was measured and ANOVA and Tukey tests were used for statistical analysis.

**Results:**

No statistically significant differences were observed between different preparation techniques. The intact roots showed significantly greater fracture resistance compared to both instrumented groups (P<0.01). Resilon Group showed significantly higher resistance than gutta-percha Group (P<0.01); however the difference between Resilon and intact teeth was not statistically significant.

**Conclusion:**

Accoding to the results of this in vitro study, root canal filling using Resilon may increase the fracture resistance of treated teeth.

## Introduction

Root canal instrumentation is an important part of endodontic treatment. Excessive dentin removal through instrumentation makes the root structure weaker and increases its potential to fracture [1, 2]. Most vertical root fractured teeth end up being extracted or if possible removal of the fractured root in the case of multi-rooted teeth occurs[3].

Currently, there are two major conservative methods for tooth preservation including less possible intraradicular dentin removal and minimizing intracanal wedging forces [4]. In addition, utilizing materials which can reinforce root tooth structure may be beneficial [5]. Bonding of an endodontic material to intracanal dentin might possibly improve resistance to fracture of endodontically treated teeth. In order to achieve this goal, glass ionomer-based sealers have been suggested for root canal obturation [6, 7]; but, glass ionomer is technique sensitive and also hard to remove if further treatment is needed [6].

A relatively newer adhesive obturation system named Resilon and Epiphany has also been used to improve resistance to fracture of endodontically treated teeth [7]. Resilon used for root canal system obturation is handled similarly to gutta-percha. This material can be laterally condensed, as well as heat softened and injected into the root canal system [8]. A dual cure, resin based sealer (Epiphany), is used in conjunction with Resilon. It has been shown that Epiphany bonds to the dentin walls and Resilon core [9, 10]. Resilon/Epiphany system is able to penetrate into dentine tubules and provides a monoblock state obturation [4, 11, 12].

The complete removal of infected dentin and tissue by enlarging the root canal system is the main purpose of endodontic treatment. During the past decade, the advent of nickel-titanium (NiTi) rotary instrumentation has been one of the most prominent changes in root canal therapy, and it allows easier, faster, and better root canal shaping [13]. Some authors have suggested that greater taper instruments to prepare root canals may reduce the fracture resistance of the root [14]. Moreover producing rounder canal preparations and smoother canal walls [15, 16] may enable low and uniform stress distribution around the walls of canal [17]. Rotary instruments and increased taper files could have an effect on fracture resistance of roots; however, this is still a controversial issue [14, 17, 18].

The aims of this *in vitro* study were *1)* to compare fracture resistance of teeth which were obturated with gutta-percha (AH26 sealer) with those obturated with Resilon and Epiphany obturation system; and *2)* to evaluate and compare the effect of Race rotary files and manual stainless steel files root canal preparation on root fracture.

## Material and Methods

This study was approved by the Ethics Committees of Dental Research Center of Shahid Beheshti Medical University, Tehran, Iran, and Qazvin Medical University, Qazvin, Iran. Total of 105 single-canal extracted maxillary human incisors of patients aged 50-60 years were selected. Selected tooth samples all had periodontal problem and developed apical foramens without previous endodontic treatment, caries or root resorption. All teeth were examined under a dental operating microscope (Olympus, Tokyo, Japan) with ×20 magnifications to detect any minute crack or fracture, and root length was adjusted to 16 mm from CEJ to apex. Canal curvature was less than 15°. All debris and remaining tissues were removed using hand scalers. For disinfection, samples were stored in 5.25% sodium hypochlorite (Household Bleach golrang, Tehran, Iran) for 1 hour; then we placed them in normal saline before the experiment. Teeth were decoronated at the CEJ level using a Diamond Disc (Jota AG, Zurich, Switzerland) attached to laboratorial handpiece.

Root length was established by manually inserting #15 K-files (Dentsply Maillefer, Tulsa, Ok, USA) into the canals, until the file tip was visible at the apical foramen. Working length was determined 1.0 mm shorter than real root canal length. All teeth, except those in control group, were instrumented using either manual stainless steel files (Dentsply Maillefer, Switzerland) or rotary Race files (FKG, Dentaire Co., Dental Products, Switzerland).

In the manual instrumentation group, apical preparation of canals was carried out with stainless steel files to a #40 K-file as a master apical file, then #2 and 3 gates glidden drills (Dentsply, Maillifer, Switzerland) were used to widen the coronal two third. Patency was obtained with a #15 K-type file. Canals were irrigated with 10 mL of 5.25% NaOCl. To remove the smear layer, 3 mL of 17% EDTA (Ariadent, Tehran, Iran) were introduced and allowed to remain in the canals for 3 minutes. Then, a final flush 1 mL of 5.25% NaOCl followed by 5 mL of normal saline was performed.

In the rotary group, the root canal systems were instrumented to the working length using crown-down technique by RaCe rotary system up to #40 (0.04). Preparation was carried out by Endo IT motor (VDW, Munich, Germany) at 300 rpm. Irrigation was performed the same as manual instrumentation group. Samples were then dried with sterile paper points (Gapadent Co., LTD, Korea) and were reexamined microscopically (×20 magnification) to visualize cracks. Teeth were randomly divided into seven experimental groups of 15 each. The obturation for each group was conucted following manufacturer's instructions using NiTi finger spreaders (Dentsply, Maillefer, Switzerland). The obturation material was removed in all groups up to 2 mm apical to the orifice and cervically sealed with Coltosol (AriaDent, Tehran, Iran).

In group 1, teeth were prepared manually, and then obturated using lateral compaction technique with gutta-percha (Gapadent Co., LTD, Korea) and AH26 sealer (DeTrey, Dentsply, Konstanz, Germany).

In group 2, teeth were prepared manually and obturated with Resilon (Epiphany; Pentron Clinical Technologies, Wallingford, CT, USA).

In group 3 and 4, samples were prepared with Race rotary system, and obturated and sealed the same as group 1 and 2, respectively.

In group 5 and 6, samples were prepared with the same method as group 1 and 3, respectively, but were not obturated and were only sealed with Coltosol.

In the control group, (intact teeth) the teeth were not prepared or restored.

Finally, all samples were inspected by means of periapical radiography in order to detect any defect or crack; all roots were stored at room temperature in 95% humidity for one week to allow complete setting of the sealers.

All prepared teeth were vertically set in self-cure acrylic resin (Bayer, AG, Germany) within the rings that had height of 20 mm and diameter of 40 mm. The apical 8 mm of each root was kept exposed. After 24 hours, the acrylic resins were set and the blocks were stored in 95% humidity before mechanical tests. Universal testing machine (Zwick GM 2010, Zwick roell, Germany) was used for mechanical examination. The upper part of the machine housed a round tip of 4 mm diameter that was placed in contact with the occlusal surface of the sample. Compressive loading was applied at a crosshead speed of 1 mm/min until fracture occurred. The measured value at fracture, which was recorded as fracture strength of specimen, was recorded in Newtons (N). Data were statistically analyzed using one way ANOVA and Tukey’s HSD. Results with *P*<0.01 were considered significant.

## Results

Significant differences were observed between the loads, which fractured the teeth (Table 1). No statistically significant differences were observed between the rotary (group 3, 4) and teeth that were prepared manually (group 1, 2). Also, there was no statistical significant difference between the teeth that were obturated with gutta-percha and un-restored teeth.

**Table 1.** Mean (SD) fracture resistance values of root canals in experimental and control groups

	Gutta-Percha	Resilon	Not obturated
**Hand instrumentation**	368.76 (94.37)	569.95 (123.60)	355.17 (77.38)
**Rotary**	375.52 (71.96)	599.81 (87.76)	333.07 (73.89)
**Intact teeth**			618.53(123.72)

The intact roots showed significantly greater fracture resistance compared to both instrumented groups (*P*<0.01). Resilon group showed significantly higher resistance than gutta-percha groups (*P*<0.01); while the difference between Resilon group and intact teeth was not statistically significant.

## Discussion

It is well established that the preparation of the root canal system removes significant amount of tooth substance, and that the use of unnecessary force during obturation weakens the tooth, decreasing the fracture resistance of endodontically treated teeth [19]. According to our findings, intact teeth had significantly more fracture resistance than endodontically treated roots. This was in agreement with other studies [20, 21]. Also the results of our study (Table 1) demonstrated that the instrumented but unfilled roots were significantly weaker than the obturated ones. Root fracture resistance showed 43% and 48% decrease after instrumentation with hand instruments and Race rotary files, respectively; but no statistically significant differences were observed between these two instrumentation groups.

Zandbiglari and Schafer also demonstrated that roots which were prepared with manual technique and rotary files showed lower fracture resistance compared to intact roots with no significant difference between two instrumented groups. They showed that the greater tapered roots needed less force to fracure [3]. These results concur with our study and other previous studies [5, 14].

In this study, smear layer was removed using 3.0 mL of 17% EDTA. The smear layer not only can provide an avenue for leakage [22], but also act as a barrier between root filling materials and the surface of the root canal walls [23]; it may therefore compromise the formation of a monoblock.

As previously mentioned, an ideal root canal filling material should be able to reinforce and strengthen a weakened root structure against fracture in addition to sealing the canal. Although, gutta-percha as an endodontic root filling material is the golden standard, limitations such as coronal microleakage and inability to reinforce endodontically treated roots have led to the introduction of some new products [24]. The Resilon/Epiphany system provides a new obturation material for endodontic treatment. This system creates a chemical bond with root canal structure that is maintained over time; therefore, representing a better option than gutta-percha [25-27]. Resilon is a synthetic polymer, and thus, resin sealer attaches to it as well as to bonding agent or primer. Furthermore, primer penetrates easily into dentinal tubules. In so doing, a monoblock is formed (consisting of Resilon core material, resin sealer, bonding agent/primer and dentin) [21, 28].

According to the present study, in comparison to root canals that were obturated with Resilon/Epiphany system, other specimens except the control group showed less fracture resistance agreeing with other studies [3-5, 29, 30]. This could be due to the Resilon/Epiphany system chemical bond with tooth dentine. Several studies have shown that chemical bonding to root dentin enhances the resistance of endodontically treated teeth against root fractures [4, 11, 31].

## Conclusion

Within the limitations of the present *in vitro *study, it can be concluded that Resilon has the potential to enhance the root fracture resistance in endodontically treated teeth. In addition, manual and Race rotary preparation methods have similar effects on root fracture resistance.
